# Supplementary figures and images for: Elastic Strain Relaxation of Phase Boundary of α′ Nanoscale Phase Mediated via the Point Defects Loop under Normal Strain
Source: Nanomaterials (Basel). 2023 Jan 22;13(3):456. doi: 10.3390/nano13030456 (PMC9920593; doi:10.3390/nano13030456)

## Slide 1
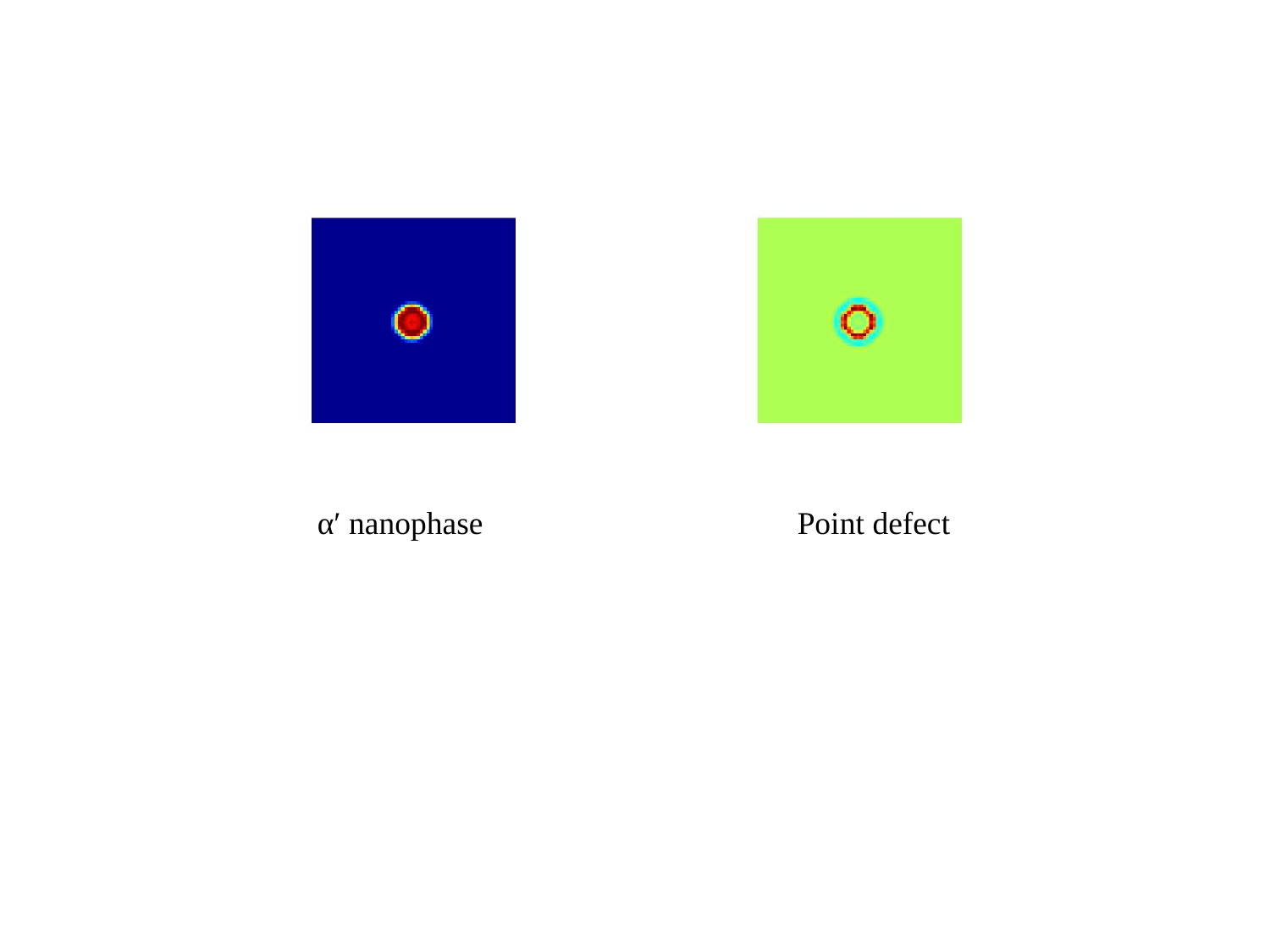

α′ nanophase
Point defect

Supplement: Supplementary file 1 [file nanomaterials-13-00456-s001.zip › nanomaterials-2079806-supplementary.pptx]
